# Matrix Formalism to Describe Functional States of Transcriptional Regulatory Systems

**DOI:** 10.1371/journal.pcbi.0020101

**Published:** 2006-08-11

**Authors:** Erwin P Gianchandani, Jason A Papin, Nathan D Price, Andrew R Joyce, Bernhard O Palsson

**Affiliations:** 1 Department of Biomedical Engineering, University of Virginia, Charlottesville, Virginia, United States of America; 2 Department of Bioengineering, University of California San Diego, La Jolla, California, United States of America; 3 Bioinformatics Program, University of California San Diego, La Jolla, California, United States of America; Pennsylvania State University, United States of America

## Abstract

Complex regulatory networks control the transcription state of a genome. These transcriptional regulatory networks (TRNs) have been mathematically described using a Boolean formalism, in which the state of a gene is represented as either transcribed or not transcribed in response to regulatory signals. The Boolean formalism results in a series of regulatory rules for the individual genes of a TRN that in turn can be used to link environmental cues to the transcription state of a genome, thereby forming a complete transcriptional regulatory system (TRS). Herein, we develop a formalism that represents such a set of regulatory rules in a matrix form. Matrix formalism allows for the systemic characterization of the properties of a TRS and facilitates the computation of the transcriptional state of the genome under any given set of environmental conditions. Additionally, it provides a means to incorporate mechanistic detail of a TRS as it becomes available. In this study, the regulatory network matrix, **R**, for a prototypic TRS is characterized and the fundamental subspaces of this matrix are described. We illustrate how the matrix representation of a TRS coupled with its environment (R*) allows for a sampling of all possible expression states of a given network, and furthermore, how the fundamental subspaces of the matrix provide a way to study key TRS features and may assist in experimental design.

## Synopsis

Complex regulatory networks control the transcription state of a genome that defines the components of a biochemical network. These transcriptional regulatory networks have been mathematically described. The purpose of many such mathematical models is to allow for the prediction of gene expression under a variety of environmental conditions. However, to date, quantitative models have been limited in scope due to a paucity of relevant data, and models of larger networks have been limited in their quantitative predictive power. Herein, Gianchandani and colleagues present a formalism that represents regulatory rules in a matrix form which attempts to address these issues. This matrix formalism allows for the systemic characterization of the properties of a transcriptional regulatory system and facilitates the computation of the transcriptional state of the corresponding genome under any given set of environmental conditions. Additionally, it provides a means to incorporate mechanistic detail of a transcriptional regulatory system as it becomes available. The authors illustrate how this matrix representation allows for a sampling of all possible expression states of a given network and provides a way to study key features. They also present how it may assist in experimental design to interrogate genome-scale cellular networks.

## Introduction

With the delineation of multiple genome sequences, there is an increased interest in understanding how the genes within a given genome are regulated through complex transcriptional regulatory networks (TRNs). Consequently, there is an effort under way to reconstruct the TRNs of model organisms [1]. Because the number of regulated genes and associated regulatory proteins is quite large and their interconnectivity is extensive, there is a significant need for a structured framework to integrate regulatory rules and interrogate TRN functions in a systematic fashion. Such a framework should generate hypotheses for experimental investigation to further characterize a given regulatory program.

Several approaches have been used to characterize features of TRNs, including Bayesian networks [2], Boolean networks [3–6], and stochastic equations [7] (see [8] for a review of many such methods). While most of these methods have been applied to relatively small systems due to a lack of relevant data, there are notable exceptions (for examples, see [9–12]). Two of these are briefly described. First, a reconstruction of the regulatory network that controls sea urchin development has been formulated, and the temporal profile of 40 genes involved in the embryogenesis of the sea urchin characterized [12]. Second, an integrated analysis of metabolic and regulatory networks in Escherichia coli was performed [9] through dual perturbation experiments [13]. This systematic approach to reconstructing and interrogating the integrated network of E. coli led to the novel characterization of multiple regulatory rules and an expansion of a genome-scale TRN, based on a model-driven analysis of multiple high-throughput datasets.

Although the components and component interactions of large-scale TRNs have been reconstructed, the properties of the functional states of these networks have not yet been extensively investigated. Consequently, there is a need for a structured, self-contained representation of TRNs that can be quantitatively interrogated. This paper presents a novel approach for describing a complete transcriptional regulatory system (TRS), including inputs and outputs to the set of internal reactions defined by the TRN, in a functional matrix form (called a regulatory network matrix, or **R**) that connects environmental cues to transcriptional responses. It can be used to compute the expression (i.e., functional) state of the TRS that it represents. To illustrate this approach, the regulatory network matrix for the *lac* operon TRS in E. coli is characterized and the fundamental subspaces of the matrix are described. Furthermore, this matrix representation of transcriptional regulation is used to efficiently sample all possible expression states of a prototypic TRS.

## Materials and Methods

### Conceptual Framework

**Figure 1:**
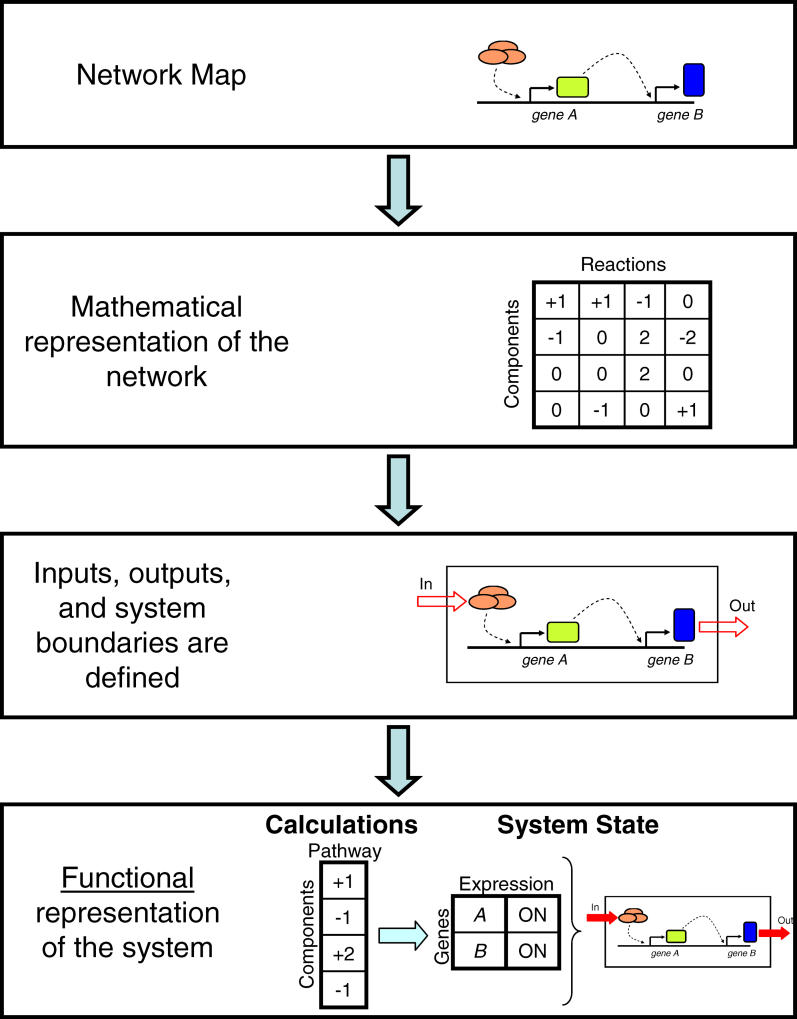
Toward a Functional Description of a Biological System The general process of connecting a network map to a functional description of the network is depicted. A network is comprised of components and the interactions between them, and it is often graphically displayed as a map. This map is in turn represented mathematically as an incidence matrix that captures the stoichiometry of the underlying chemical transformations of the network. Subsequently, the boundaries of the network and its inputs and outputs are delineated, yielding a complete system. Finally, matrix analysis techniques are used (e.g., extreme pathway analysis) to generate functional descriptions of system properties (e.g., system states).

The process of converting a network map to a functional description of the network is depicted in Figure 1. Networks comprise components and interactions between them, often graphically displayed as maps that illustrate the relationships between the input state of a network and its corresponding output state. Maps can be represented mathematically as incidence matrices [14]. If the underlying chemical reactions of the network can be delineated, the network can be described with a stoichiometric matrix, **S**, that captures the reaction stoichiometries [15]. Once the network boundaries are defined, inputs and outputs are delineated and a system is defined. Matrix analysis methods can then be used to generate functional descriptions of network properties and states [16]. (For background about the generation of a stoichiometric reconstruction as well as the associated analysis techniques as previously reported and applied to metabolic and signaling networks, see also Protocol S1.) In the following sections, we describe how regulatory interactions can be represented in a similar fashion to **S** using a regulatory network matrix, **R**, to describe a TRS.

### The Formation of R and the System It Represents

**Figure 2:**
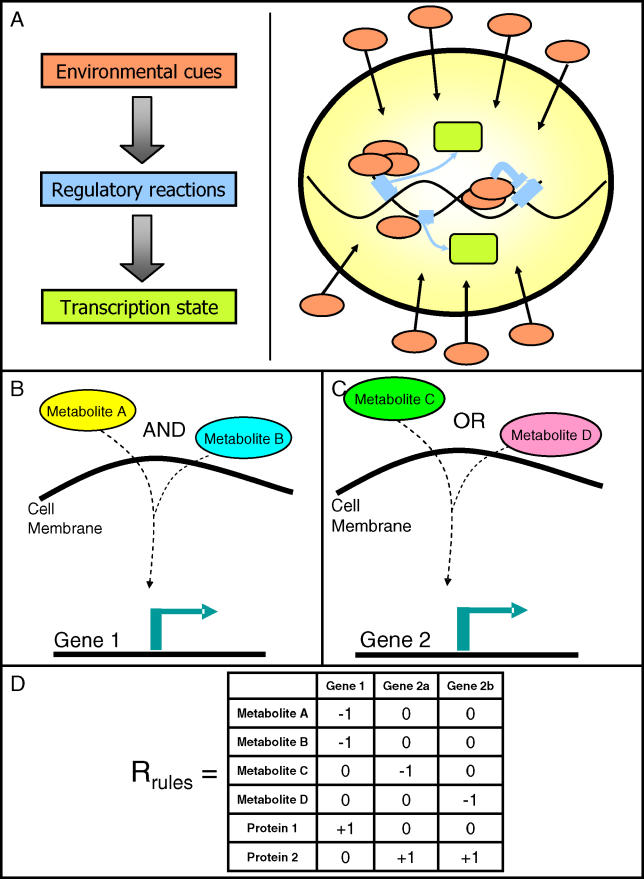
The Formation of the TRN Matrix (A) Depicts the TRS as a biological system consisting of inputs, internal reactions, and outputs. The inputs are environmental cues, including the presence and absence of metabolites, reaction fluxes, and specific conditions. The internal reactions are the regulatory rules that describe the activation or inhibition of gene transcription. The output is the transcription state, which is a collection of the protein products that result through a combination of the environmental cues acting upon the regulatory rules. (B) Depicts a situation where the presence of both of two metabolites is required for the expression of *Gene 1*. (C) Depicts a situation where the presence of either of two metabolites leads to the expression of *Gene 2*. (D) The associated regulatory network matrix (**R_rules_**) represents each of these two regulatory rules of the sample TRN in quasi-stoichoimetric formalism. *Gene 1* requires the presence of *Metabolite A* and *Metabolite B,* and the output of the reaction is the associated protein. *Gene 2* requires either *Metabolite C* or *Metabolite D,* and the product is the associated protein. The rule associated with the activation of *Gene 2* can be satisfied with two independent conditions. Thus, there are two columns corresponding to the activation of *Gene 2*.

The **R** matrix describes the connections between environmental cues and transcriptional responses. This relationship is illustrated in Figure 2. Figure 2A shows the typical depiction of a TRS as a biological system comprising a collection of inputs, internal reactions that form the TRN, and outputs. In general, the inputs are environmental cues, including the presence and absence of extracellular metabolites, reaction fluxes, and specific conditions such as certain pH values. The internal reactions, often not known in chemical detail, are represented by regulatory rules that describe the activation or inhibition of gene transcription in response to environmental cues. The outputs are the synthesized protein products that result through a combination of the signaling inputs acting upon the regulatory rules as well as consequent transcription and translation. A representative pair of regulatory rules is presented in Figure 2B and 2C. In Figure 2B, the expression of *Gene 1* depends on the presence of both *Metabolite A* and *Metabolite B*. The presence of both metabolites is required for the transcription of the associated gene. In the example provided in Figure 2C, the transcription of *Gene 2* depends on the presence of either *Metabolite C* or *Metabolite D*. Accordingly, the presence of either metabolite can lead to the transcription of the corresponding gene.

**Figure 3:**
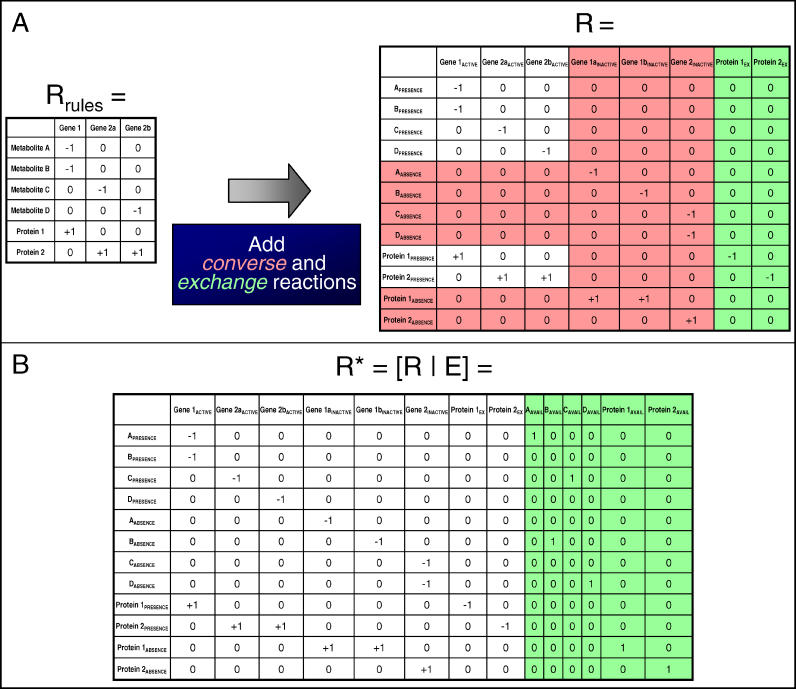
The Matrix as a Representation of a TRS (A) Depicts the TRN matrix shown in Figure 1 (**R_rules_**). This matrix is expanded to yield the matrix **R**, which describes the complete TRS. The **R** matrix includes the converse of the regulatory rules, i.e., the regulatory reactions that lead to the *inhibition* of gene transcription (rows and columns shaded in pink). For example, the inhibition of *Gene 1* requires either *Metabolite A* or *Metabolite B* to be absent. The **R** matrix also includes exchange reactions that balance the production of proteins and enforce the fact that the protein products are outputs of the TRN (columns shaded in green), thus representing a TRS. (B) The TRS **R** matrix is combined with the environment matrix (**E**), which consists of a set of columns that represent a particular environment of signaling stimuli (columns shaded in green), to yield the matrix R*. The presence or absence of a given signaling stimulus is denoted by a 1 or a 0 in the corresponding row in **E**. Initially, all the proteins are absent, and the availabilities of these are updated as the matrix is analyzed. Ultimately, these columns in **E** indicate the activation or inhibition of gene transcription for the environment.

These relationships can be represented in a matrix of regulatory rules (**R_rules_**), which is a subset of the complete **R** matrix (discussed below). In Figure 2D, the regulatory rules associated with *Gene 1* and *Gene 2* are represented as three columns (or regulatory reactions) of the matrix. The four metabolites are indicated as separate rows. The metabolite-reaction relationships are represented in a quasi-stoichiometric formalism. Here, *quasi-stoichiometric* indicates that each column of the matrix accounts for the relationship between regulators and the genes that they control without necessitating mass balance. Conceptually, the regulators are “consumed” and the gene products are produced. Importantly, this formalism can account for mass-balanced relationships in TRSs [17]. For example, the transcription of *Gene 1* depends on both *Metabolite A* and *Metabolite B*. Consequently, we define the inputs to the *Gene 1* reaction (*Metabolite A* and *Metabolite B*) as −1, and the output from the reaction (Protein 1) as +1.

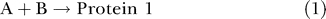
For the expression of *Gene 2,* either *Metabolite C* or *Metabolite D* can function to activate transcription. Consequently, there are two independent columns to represent the regulatory rule associated with the activation of *Gene 2*.

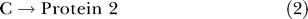


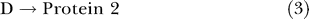
This matrix can be used to represent a TRN (Figure 3). In Figure 3A, the regulatory network matrix consisting of regulatory rules from Figure 2 (**R_rules_**) is indicated.

To convert the TRN into a TRS, the matrix of regulatory rules is expanded to include: 1) the converse of the regulatory rules, and 2) exchange reactions that balance the production of proteins. The resulting system, with inputs, outputs, and internal reactions, is represented by the complete regulatory network matrix called **R**. The process of forming a TRS from the TRN requires more explicit description, as we have to account for the “absence” of network components and their “exchange” with the environment.

1) The *converse* of the regulatory rules (i.e., the regulatory reactions that lead to the inhibition of gene transcription in our sample system) is necessary to reflect the lack of protein production for a given set of environmental cues (Figure 3A). Many regulatory rules are inhibitory, such that the expression of a protein depends on the absence of a given metabolite or protein product. Additional rows for the absence of metabolites and protein products as well as columns representing the converse of the regulatory rules are included. Again, these relationships are represented in quasi-stoichiometric formalism. For example, the converse of the regulatory rule for *Gene 1* implies that the transcription of *Gene 1* is inhibited if either *Metabolite A* or *Metabolite B* is absent. Consequently, there are two independent columns (*Gene 1a_INACTIVE_* and *Gene 1b_INACTIVE_*) to represent the reactions for the regulatory rule associated with the inhibition of *Gene 1*.

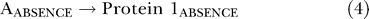


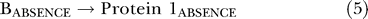
Similarly, the inverse of the regulatory rule for *Gene 2* implies that the transcription of *Gene 2* is inhibited if *Metabolite C* and *Metabolite D* are both absent. Consequently, we define the inputs to the *Gene 2_INACTIVE_* reaction (absence of *Metabolite C* and absence of *Metabolite D*) as −1, and the output of the reaction (absence of Protein 1) as +1.


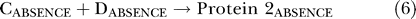


2) Further, the quasi-stoichiometric formalism needs to be supplemented by *exchange* reactions that balance the production of proteins (Figure 3A). These exchange reactions describe the role of the proteins as outputs of the TRS. Once they are produced, the proteins can exit the TRS and perform their associated cellular tasks. Therefore, columns representing the exchange of proteins are incorporated. These columns have an entry of −1 in the corresponding row to indicate that the protein leaves the TRS (i.e., it is “depleted” from the TRS).

The TRS will respond to environmental signals, whose state (i.e., presence or absence) needs to be specified. In Figure 3B, the **R** matrix is further combined with the environment matrix (**E**), which characterizes the environment against which a set of regulatory rules is to be evaluated, yielding R*. The columns of the **E** matrix denote the availability of metabolites and protein products. For the sample environment shown, the presence of *Metabolite A* is indicated by a +1 in the row “*A*_PRESENCE_” and the column “*A*_AVAIL_,” whereas the absence of *Metabolite B* is indicated by a +1 in the row “*B*_ABSENCE_” and the column “B_AVAIL_,” and similarly for *Metabolite C* and *Metabolite D*. Initially, for any environment, the proteins that are part of the TRS are assumed to be absent, and the values of these columns are subsequently updated as **R** is analyzed in silico. Just as the continuous production of proteins (the outputs from the TRS) is balanced by exchange reactions, the columns representing a particular set of environmental cues serve to balance the set of environmental cues. Thus, these exchange reactions describe the role of the environmental cues as inputs to the TRS. Once they enter the TRS, they can participate in the regulatory reactions and initiate gene transcription. Since the compounds in **R** must be balanced with the environment in **E**, the resultant matrix R* is used to calculate expression (i.e., functional) states of a TRS. In a similar fashion, the analysis of the functional states of stoichiometric networks through **S** needs the definition of exchange reactions and their associated connections with a particular environment [18].

### Analysis of Functional States of a TRS

Many methods for analyzing genome-scale stoichiometric matrices have been developed and used to gain biological insight [16,19–24]. One such approach is called extreme pathway analysis [25]. Extreme pathway analysis has previously been applied to metabolic and signaling networks to determine the set of systemically independent pathways through a network [26–28]. Briefly, the extreme pathways are a minimal and unique set of generating vectors that define the edges of the convex solution space that contains all valid steady-state flux distributions in a network. Any possible solution or flux distribution can be described as a non-negative linear combination of these extreme pathways. In effect, the extreme pathways span a convex space that circumscribes all potential functional states (i.e., phenotypes) of a network.

Analogously, for a TRS, extreme pathway analysis yields a set of generating vectors that encompasses all possible expression states of the network. Consequently, extreme pathway analysis represents an in silico technique for evaluating global characteristics of gene expression. The extreme pathways are a set of systemically independent, convex basis vectors. As such, they represent the extreme states of the TRS; any possible expression state of a TRS is a non-negative combination of these basis vectors. Following the formalism previously developed [25], the internal regulatory reactions, or relationships, can only have non-negative weights, while the environmental reactions, or relationships, may have values of −1 or +1 to represent the absence or presence of a given component, respectively. As described below, the pervasiveness of signal inputs, percentage of environments in which a given gene is expressed, numbers of genes expressed together, and correlated gene sets represent the type of data that can be readily generated for a TRS by analyzing R* in different environments using this approach.

### The Four Fundamental Subspaces of R*

The four fundamental subspaces, namely the null space, left null space, row space, and column space, describe key properties of a matrix, and consequently the system that it represents. For a stoichiometric matrix that represents a biological network, these fundamental subspaces represent key system properties [18]. Singular value decomposition (SVD) is used to decompose a matrix into three matrices, often referred to as **U**, **Σ**, and **V** [29], that delineate the four fundamental subspaces (see [29] and [18]).

Each of these four fundamental subspaces contains particular information about the original matrix. A vector **e** that satisfies the equation (R*)•**e** = **0** lies in the null space. Every such vector, therefore, can be multiplied into all rows of R* (i.e., each metabolite, transcription factor, and protein product of the TRS) and yield zero. For this result to be the case, the relationship between each metabolite, transcription factor, and protein product of the corresponding TRS must be conserved within the TRS as represented in the vector **e**. Consequently, each vector **e** that is part of the null space represents a balance for a given network component (row of the matrix) between the internal regulatory network (or TRN) and the environment. Therefore, a given vector **e** is the collection of active/inactive genes (columns of the matrix) that balance the TRN with the environmental cues (see [27] for further description of similar pathways, as seen in the JAK-STAT signaling pathway of the human B cell). Thus, these pathways represent link-neutral states of the TRS; each internal component (row) has an input reaction and an output reaction. The null space of R* captures all possible balanced expression (i.e., functional) states of the TRS that it represents.

For the equation (R*)^T^•**u** = **0**, the set of vectors **u** that satisfy the equation lie in the left null space, where (R*)^T^ is the transpose of R*. The multiplication of each row of (R*)^T^ (or conversely each column of R*) by a given vector **u** yields zero. Consequently, each vector **u** represents an invariant pool of network components (rows of R*) across all component interactions (columns of R*). Therefore, the left null space of R* contains pools or aggregates of network components that are invariant across all the regulatory rules of the TRS. Thus, these pools represent node-neutral states of the TRS; each internal reaction or relationship (column) has an input node and an output node. For example, in detailed reconstructions, these pools represented by **u** may be groups of open reading frames that are coordinately regulated and can be classified as regulated units, or regulons.

The columns of R* (that are vectors in the column space of R*) contain information regarding the similarity or difference between how genes (and the corresponding protein products) are regulated. For example, a small angle between a pair of columns (i.e., the corresponding vectors) in R* indicates that the regulatory rules of the two corresponding genes are very similar and affect the state of the TRS in a similar fashion. Conversely, a large angle between a pair of columns in R* indicates that the genes are regulated by very different sets of rules. Note that, for any given gene, multiple columns may be required to capture different parts of a complex Boolean regulatory rule.

The rows of R* (that are vectors in the row space of R*) contain information about the overall similarity of network component participation in the generation of expression states. For example, for the rows corresponding to environmental cues, the row vectors describe the influence that these environmental cues have in generating an expression state. A small angle between a pair of these rows in R* indicates that the corresponding environmental cues have very similar effects on the expression state of the network. Conversely, a large angle between a pair of these rows in R* indicates that the corresponding environmental cues have very different effects on the expression state of the network. Although not investigated in the present study, the further exploration of this concept may yield important insight into how a TRS moves from one functional state to another.

These properties contained within the fundamental subspaces of R* can translate to experimental design. For example, in selecting a set of environmental conditions to probe the functions of a given TRS, the set of inputs which generate very different effects on the expression state of the TRS would provide the most information in a given experiment. These fundamental properties of R* are described below in more detail in the context of the *lac* operon in *E. coli,* as well as in the context of the TRS for a prototypic system that emulates key features of prokaryotic TRSs.

## Results

The matrix formalism for representing TRSs was evaluated using a small-scale reconstruction of the TRN of the *lac* operon in E. coli. Furthermore, a larger prototypic TRN was constructed to reflect the types of transcriptional regulatory mechanisms observed in a previous reconstruction of the genome-scale E. coli TRN [9]. Ultimately, the ability of the framework to incorporate mechanistic detail as it becomes available on the genome scale is illustrated.

### An Example System: The Regulatory System of the *lac* Operon in E. coli

**Figure 4:**
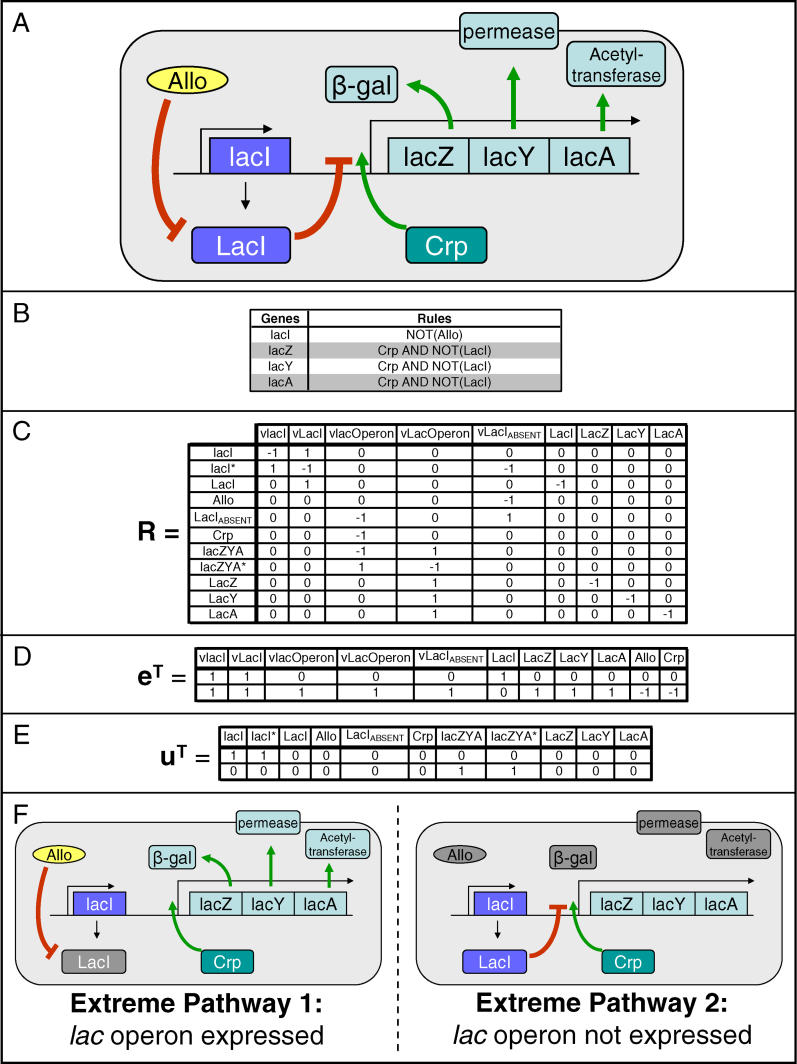
The TRS for the *lac* Operon in E. coli (A) The system is defined to include the *lac* operon genes (*lacZ, lacY, lacA*), the inhibitor gene *lacI,* the activator Crp, and the inducer allolactose (*Allo*). (B) Summarizes the Boolean rules that capture the regulatory logic of the system. (C) The **R** matrix is shown, with each row corresponding to system components and each column specifying regulatory reactions in a quasi-stoichiometric formalism. Accordingly, a “−1” represents a consumed component, whereas a “+1” represents a produced component. (D and E) Depict the null space and left null space, respectively. (F) The two extreme pathways from the null space in (D) are presented. Pathway 1 illustrates the conditions for the activation of the *lac* operon (i.e., inhibition of LacI by allolactose, thus allowing for Crp-activated expression of *lacZYA*), whereas pathway 2 illustrates the conditions for the LacI-mediated inhibition of the *lac* operon.

In an effort to explore this modeling framework and to assess potential challenges, the TRN that dictates the expression of the *lac* operon in E. coli was modeled (Figure 4). For the purpose of this investigation, the system is defined to include the *lac* operon (*lacZYA*) and the proteins that each operon gene encodes; the inhibitor of the operon (*lacI*); an activator of the operon (Crp); and the intracellular inducer molecule allolactose, which inhibits the LacI inhibitor thus activating *lacZYA* transcription (Figure 4A).

Having defined the system and Boolean rules that specify the regulatory logic of this TRN (Figure 4B), the TRS can be formulated and the associated **R** matrix constructed (Figure 4C). As previously described, each row in **R** describes a TRS component (i.e., gene, metabolite, transcription factor, or protein product), and each column specifies a regulatory event (i.e., reaction). For the purposes of this analysis, each gene/operon is depicted within the matrix twice: *lacI* and lacI*, as well as *lacZYA* and lacZYA*. The former entity represents the open form, whereas the latter, asterisk-marked entity, represents the actively transcribed form of the gene. This level of detail is not required in formulating **R** as the actively transcribed form of the gene is only a transient entity between transcription and translation. However, as such mechanistic detail about open reading frames and other network relationships becomes available for actual TRSs, the formalism presented herein can readily incorporate it.

Null space vectors **e** that satisfy (R*)•**e = 0** and left null space vectors **u** that satisfy (R*)^T^•**u** = **0** were then calculated for the defined *lac* TRS. All possible network expression states are defined by the two vectors that span the null space (Figure 4D). (It is important to note that, in calculating these expression states, it was assumed that Crp is a part of the TRS and always present whereas allolactose is a part of the TRS but variable by environment. This assumption was made to avoid accounting for the specific regulation of Crp production in order to maintain the relative simplicity of this example. In other words, only two possible environments were evaluated, one in which allolactose is present and another in which it is absent.) These vectors are the extreme pathways of the TRS. For reaction names prefaced with a “v,” a 1 indicates that the reaction is active, and a 0 indicates that it is inactive. In the remaining reactions that specify flow across the system boundary, a 1 indicates flow out of the system (for example, a protein is produced), a −1 indicates flow into the system, and a 0 indicates that the associated component is neither produced nor consumed. Note that these entries denote an active connection, and series of connections lead to a causal path. The first vector represents the LacI-mediated inhibition of the *lac* operon. The second vector defines the inhibition of LacI by allolactose, thus allowing for Crp-activated expression of *lacZYA*. These two vectors thus represent the two expression states of the *lac* operon system, and they are further depicted graphically in Figure 4F.

Analysis of the left null space identified two intra-network pools in the defined *lac* TRS, and these are represented by two convex vectors that represent the extreme states of the TRS, calculated by determining the extreme pathways of the transpose of R* (Figure 4E). In the vectors specified for **u**, a 1 represents that the system component denoted by the column header in the pool is present, and a 0 indicates that the system component is absent from the pool. The pool depicted by the first vector specifies the *lacI* gene pool, as the open (*lacI*) and actively transcribed (lacI*) forms of the gene together represent a conserved quantity within the system. Likewise, the second vector describes the conserved *lac* operon pool by specifying the lacZYA–lacZYA* conserved quantity. Again, these conserved quantities are the extreme states of the TRS. For the *lac* operon system, these two pools are relatively straightforward; *lacZYA* will be either open *or* actively transcribed. Thus, the corresponding pool represents this invariant grouping. For larger systems, these pools may be groups of open and/or actively transcribed sets of genes. Such invariant groupings may correspond to complex regulated transcriptional units, or regulons, across a genome.

Pools of open reading frames are not the only type of conserved quantities that could emerge from R*. For example, one could explicitly model the activation of a transcription factor by a small molecule or metabolite (i.e., cyclic AMP [cAMP] activation of Crp). In this case, one would expect to detect a free transcription factor/metabolite/metabolite-bound transcription factor pool within the system (i.e., following from the previously mentioned example, a Crp/cAMP/Crp–cAMP pool would be identified). This pool would emerge because the collective presence of Crp, cAMP, and Crp-cAMP would be constant. Similarly, complex groupings of metabolites and transcription factors may emerge from genome-scale TRS analysis.

### Prototypic TRS

**Figure 5:**
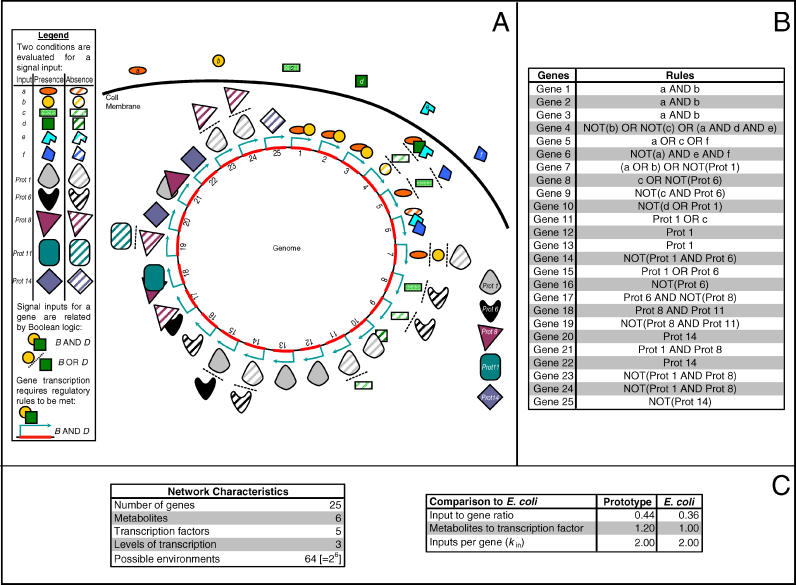
A Prototypic TRN (A) A prototypic TRN consisting of 25 genes, six extracellular metabolites, and five transcription factors, is shown. This prototype was constructed on the basis of the general characteristics of the E. coli TRN. (B) The Boolean regulatory rules that correspond to the transcription of the 25 genes within the prototypic TRN are listed. (C) Relevant characteristics of the prototypic TRS, including comparisons to the TRS of *E. coli,* are presented. This prototypic TRS gives rise to 64 [= 2^6^, where there are six inputs] possible environments, and these may be evaluated using the 64 different matrices that are generated by combining the TRS **R** matrix with each of the 64 different environment matrices (**E**).

The use of the proposed formalism for analyzing the classical *lac* operon is illuminating. The next question that arises involves evaluating how amenable this approach is to large-scale TRNs. Thus a larger prototypic TRN was assembled and evaluated (see Protocol S2 for more details) that accounts for typical features of the E. coli TRN [9]. The prototypic TRN studied below is illustrated in Figure 5A. The Boolean expressions that describe the regulatory rules for gene transcription in this prototypic network appear in Figure 5B. The prototypic TRN used in this study was composed of regulatory rules associated with the expression of 25 genes. These rules were typical of Boolean rules in the E. coli reconstruction published previously [9]. The given regulatory rule must be satisfied in its entirety for the corresponding gene to be expressed; otherwise, the gene is not expressed.

A TRS was formulated for the prototypic TRN. The set of rules for the prototypic TRS accounted for the presence or absence of six compounds (*a* through *f*) and five protein products that acted as transcription factors for other genes *(Prot 1*, *Prot 6*, *Prot 8*, *Prot 11*, and *Prot 14)*. Several comparative network characteristics of the E. coli and prototypic TRSs are provided in Figure 5C.

#### Possible functional states of the prototypic TRS.

The evaluation of all possible environments (all possible combinations of inputs) facilitated the identification and analysis of the properties of the prototypic TRS depicted in Figure 5. The prototypic TRS was sufficiently small to generate and evaluate all possible environments; however, for actual TRSs, sampling procedures may be required to generate similar characterizations. For example, Monte Carlo sampling of biochemical network function has previously generated novel results for the properties and kinetic constraints in actual metabolic networks [21,30–32].

**Figure 6:**
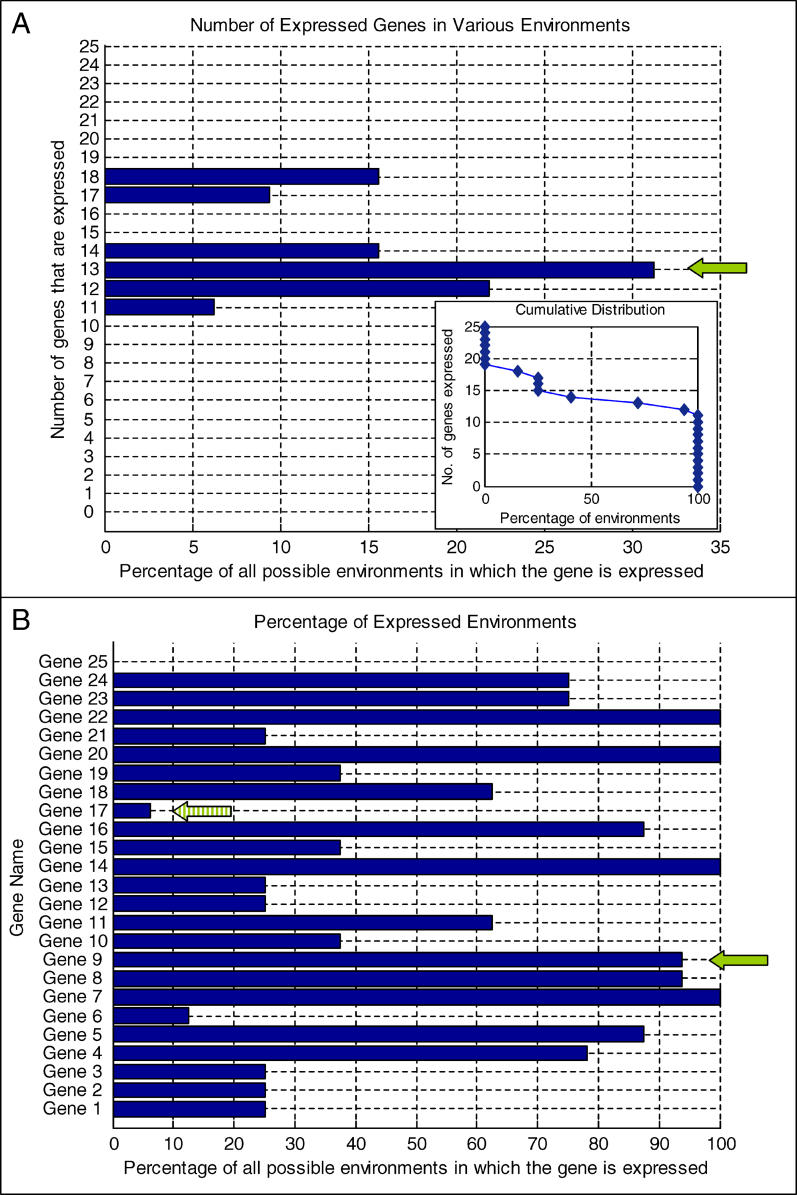
Analysis of the Prototypic TRS across All 64 [= 2^6^, Where There Are Six Inputs] Possible Environments (A) The plot indicates the percentage of all the environments (*x*-axis) in which the corresponding numbers of genes are expressed (*y*-axis). For example, 13 genes are expressed in about 31% of the environments (as indicated by the arrow). The inset shows the cumulative distribution of the percentage of all the environments in which the corresponding number of genes are expressed. Note that, for all possible environments, there are at least 11 genes and at most 18 genes expressed, and in no environments are there either 15 or 16 genes expressed. (B) The percent of environments in which the indicated gene is expressed is shown. For example, *Gene 9* is expressed in about 94% of all the possible environments (as indicated by the solid arrow). This representation of gene expression data provides insights into genes that are particularly essential or inessential for fundamental biological processes. *Gene 9,* for instance, is likely essential to cell survival and/or growth. By contrast, *Gene 17,* which is expressed in about 6% of all the possible environments (as indicated by the striped arrow), is much less essential (or its inactivation is essential) to cell survival and/or growth. These data can be used to further experimentally interrogate the system.

For the prototypic TRS in Figure 5 with six signaling inputs, there are 64 (=2^6^) possible environments (each input can either be present or absent). Thus, 64 functional, i.e., expression, states, were computed, one for each of the 64 possible environments. (A total of 64 matrices (R*) were generated by iteratively combining **R** with each of the 64 possible environment matrices (**E**), as described previously and illustrated in Figure 3B.) Figure 6A shows the percentage of these 64 environments in which particular genes are coexpressed. Most frequent is the coexpression of 13 genes, which occurs in about 31% of the environments (indicated by the arrow). By contrast, there are no environments in which fewer than 11 or more than 18 genes are expressed together. This feature indicates that the minimal set of inputs (or absence of inputs) that correspond to the expression of any genes results in the expression of 11 genes. Furthermore, for all possible inputs, no more than 18 of the 25 genes can be expressed, indicating exclusivity of certain input combinations. The inset in Figure 6A further emphasizes this point by depicting the cumulative distribution of the gene expression levels in the environments; there are zero genes that are expressed in 100% of the environments, whereas all 25 genes together are expressed in none of the environments. Such functional dependencies between environmental cues and network-wide expression states would be difficult to delineate without the structured framework that R* affords.

The percentage of environments in which a given gene is expressed was also calculated (Figure 6B). For example, *Gene 9* is expressed in about 94% of all possible environments (indicated by the solid arrow). These data offer insight into genes that may be essential for fundamental biological processes such as cell survival or cell growth. Since *Gene 9* is expressed in so many of the possible environments, these in silico expression data suggest that this gene may be necessary for critical cellular objectives. Furthermore, the expression of *Gene 9* is dependent upon the absence of both *Metabolite c* and Protein 6, and this interconnectivity within the TRS makes it difficult to identify the significance of *Gene 9* simply by looking at its regulatory rule. Since the set of all possible inputs is not indicative of the “typical” or “average” environments a given TRN may encounter, particular environments are certainly much more probable for a given TRN. The present analysis interrogates the structure of the TRS and the extreme points of all its possible states. Recent studies have demonstrated that metabolic networks occasionally operate at the extremes of their network capabilities [33,34]; thus, characterizing the set of possible states that encompass the capabilities of a network is particularly relevant.

**Figure 7:**
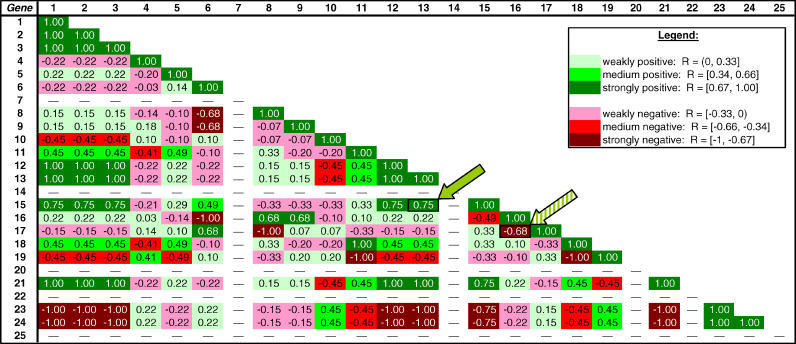
The Correlation between Gene Expression across All the Environments for the Prototypic TRS Colors indicate that the expression of two genes is correlated (green if the expression of one gene is correlated with the expression of another, red if the expression of one gene is correlated with the lack of expression of another), and the darker the color the stronger the correlation. For example, the expression of *Gene 13* is strongly correlated with that of *Gene 15* (0.75) (as indicated by the solid arrow), whereas the expression of *Gene 17* is strongly correlated with the lack of expression of *Gene 16* (and vice versa) (−0.68) (as indicated by the striped arrow). This representation identifies genes that function together versus those that function independently, and, consequently, genes that are part of the same regulated units can be identified. These correlated gene sets can therefore guide experimental design.

The set of possible expression states was also evaluated to identify the correlated presence (and absence) of groups of genes. Specifically, the correlation coefficient (*r_ij_*) between any two genes *i* and *j* in the network across all 64 environments was calculated. The matrix of pairwise correlation coefficients for all 25 genes was computed (Figure 7). Pairs of genes that are expressed together have positive correlation coefficients, whereas a pair of genes in which one gene is expressed and another gene is not expressed has a negative correlation coefficient. Genes whose expression is completely independent of each other have an *r*-value equal to zero. For example, the expression of *Gene 13* and *Gene 15* are strongly correlated (*r* = 0.75) (indicated by the solid arrow), whereas *Gene 17* and *Gene 16* are strongly anticorrelated (*r* = −0.68) (indicated by the striped arrow). Again, because regulatory rules are inherently complex (e.g., the expression of *Gene 17* is dependent upon the simultaneous presence of Protein 8 and Protein 11, whereas the expression of *Gene 16* is dependent upon the simultaneous presence of Protein 6 and absence of Protein 8), it is difficult to identify correlated gene sets without the kind of formalism and associated analysis presented herein. Correlated gene sets can describe which genes function together as well as which genes function independently and may provide insights into genes that are part of the same regulated units, much as correlated reaction sets represent reactions in metabolic and signaling networks that always appear together [27,35].

#### The null space of R*.

As described above, the extreme pathways are convex basis vectors of the null space of a matrix that satisfy constraints which ensure that the associated pathways are biologically relevant. Since the compounds in **R** must be balanced with the environment for (R*)•**e** = **0** to be satisfied, the extreme pathways must be calculated for R* (since the null space is spanned by vectors that satisfy the equation (R*)•**e** = **0**). Thus, these extreme pathways correspond to basis vectors that together describe the expression state for a given set of environmental conditions. All possible expression states for a given TRS are therefore non-negative linear combinations of these extreme pathways.

**Figure 8:**
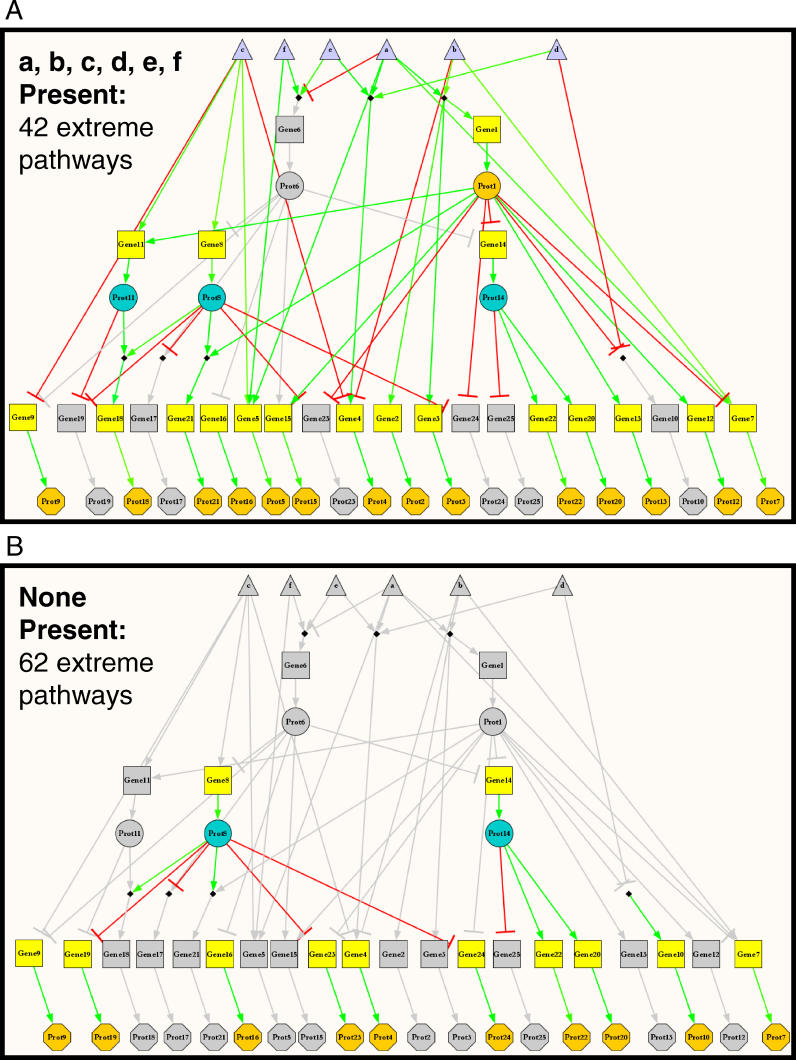
Expression States for the Prototypic TRS for the Environment in Which All Metabolites Are Present as well as the Environment in Which All Metabolites Are Absent The expression (i.e., functional) states for two different environments, as generated by extreme pathway analysis of R*, are presented. (A) The expression states for the environment in which all metabolites are present are shown. In this environment, a total of 42 extreme pathways exist leading to the activation or inactivation of the genes within the TRS. (B) The expression states for the environment in which all metabolites are absent are shown. In this environment, a total of 62 extreme pathways exist leading to the activation or inactivation of the genes within the TRS. As a legend, triangles represent extracellular cues, namely the six metabolites; squares represent the genes; and circles represent the protein products. Gray elements denote inactivity, whereas colored elements denote active components of the TRS for the given environment. Green arrows indicate that an upstream metabolite or transcription factor activates gene expression, whereas red lines indicate that the upstream metabolite or transcription factor inhibits gene expression. Lines that join together at dots denote “and” relationships within the Boolean regulatory rules, whereas lines that simply join together denote “or” relationships within the Boolean regulatory rules.

The extreme pathways were generated for each of the 64 possible R* matrices, one for each possible environment (**E**). There were 133 unique extreme pathways for the prototypic TRS (see Protocol S2 for a complete listing). The 133 extreme pathways can be grouped together to form all possible expression states of the prototypic TRS. For example, there are 42 of the 133 extreme pathways that correspond to the environment in which all six metabolites are present (see Figure 8A). Consequently, these 42 extreme pathways together constitute an expression (i.e., functional) state for the environment in which all six metabolites are present. The expression state for any particular environment (e.g., the presence of *Metabolite a* and *Metabolite b,* and the absence of *Metabolite c*, *Metabolite d, Metabolite e,* and *Metabolite f*) can be described by the combination of individual extreme pathways (a subset of the 133 extreme pathways for the prototypic TRS) that correspond to the presence or absence of the associated environmental cues.

**Figure 9:**
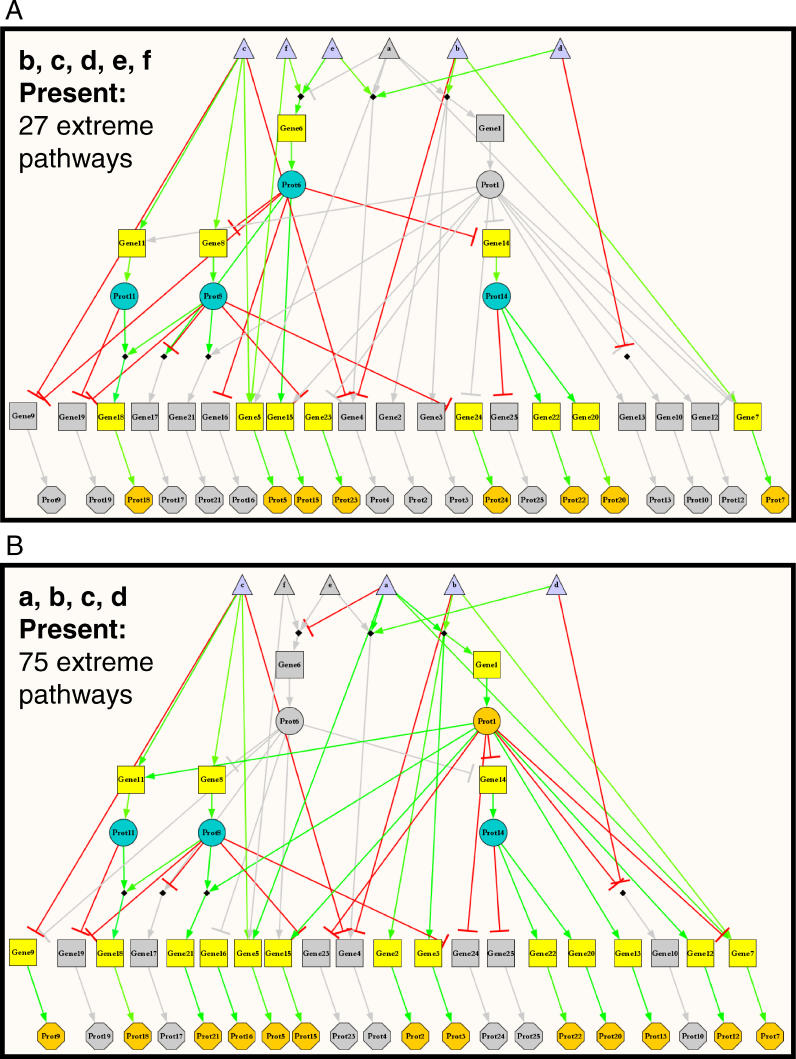
Expression States for the Prototypic TRS for Two Additional Environments The expression (i.e., functional) states for two different environments, as generated by extreme pathway analysis of R*, are presented. (A) The expression states for the environment in which Metabolite a is absent but all other metabolites are present are shown. This environment yields 27 extreme pathways, the least of all possible environments, leading to the activation or inactivation of the genes within the TRS. (B) The expression states for the environment in which *Metabolite e* and *Metabolite f* are absent but all other metabolites are present are shown. This environment yields 75 extreme pathways, the most of all possible environments, leading to the activation or inactivation of the genes within the TRS. A legend for these drawings is described in the caption for Figure 8.

The expression (i.e., functional) states of the prototypic TRS for four distinct environments, as captured through this extreme pathway analysis, are depicted in Figures 8 and 9. For example, in Figure 8A, the 42 extreme pathways corresponding to the environment in which all six metabolites are present are shown, and together they represent the expression state for that environment. Figure 8B depicts the extreme pathways that correspond to the environments in which all metabolites are absent; Figure 9A depicts the extreme pathways that correspond to the environment in which *Metabolite a* is absent but all others are present; and Figure 9B depicts the extreme pathways that correspond to the environment in which *Metabolite e* and *Metabolite f* are absent but all others are present. Different regulatory reactions occur, and different genes are activated or inactivated as a result of these interactions. In general, the portrait of complex hierarchy of gene expression that results in Figures 8 and 9 would be difficult to ascertain without this kind of analysis. The structured framework for representing TRSs presented herein can thus provide considerable insight into regulatory programs.

**Figure 10:**
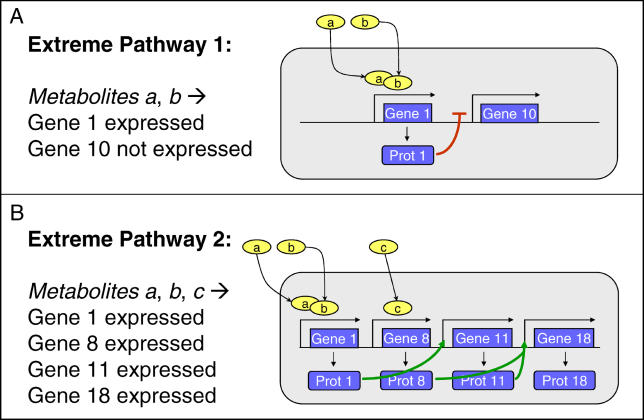
Extreme Pathways for the Prototypic TRS Two examples of extreme pathways of the prototypic TRS are highlighted for the environment in which all six metabolites (*a* through *f*) are present. In pathway 1, the presence of *Metabolite a* and *Metabolite b* activates the expression of *Gene 1,* and Protein 1*,* the product of *Gene 1,* inhibits of the expression of *Gene 10* (A). In pathway 2, the presence of *Metabolite a* and *Metabolite b* activates the expression of *Gene 1;* Protein 1 activates the expression of *Gene 11;* the presence of *Metabolite c* activates the expression of *Gene 8;* and Protein 8*,* the product of *Gene 8,* and Protein 11*,* the product of *Gene 11,* together activate the expression of *Gene 18* (B).

Another portrait of this complex hierarchy is illustrated in Figure 10, which depicts the extreme pathways of R* in two different cases. For example, in the presence of *Metabolite a, Metabolite b,* and *Metabolite c,* one extreme pathway of R* corresponds to the expression of *Gene 18* (Figure 10B). This extreme pathway consists of the terms *Gene 1, Metabolite a,* and *Metabolite b,* implying that the expression of *Gene 1* is dependent upon the presence of *Metabolite a* and *Metabolite b; Gene 8(a)* and *Metabolite c,* implying that the expression of *Gene 8* is dependent upon the presence of *Metabolite c* (specifically, the first clause of the Boolean rule for *Gene 8, Gene 8(a),* is satisfied by the presence of *Metabolite c*); *Gene 11(a),* implying that the expression of *Gene 11* is dependent upon the presence of Protein 1, the product of *Gene 1;* and *Gene 18,* implying that the expression of *Gene 18* is dependent upon the presence of Protein 8 and Protein 11, the products of *Gene 8* and *Gene 11,* respectively. In a similar fashion, extreme pathway analysis can shed considerable light on a TRS, and, when performed on the system at different time points, can evaluate other types of interactions that are not part of the prototypic TRS, e.g., regulatory loops leading to oscillations in gene expression.

**Figure 11:**
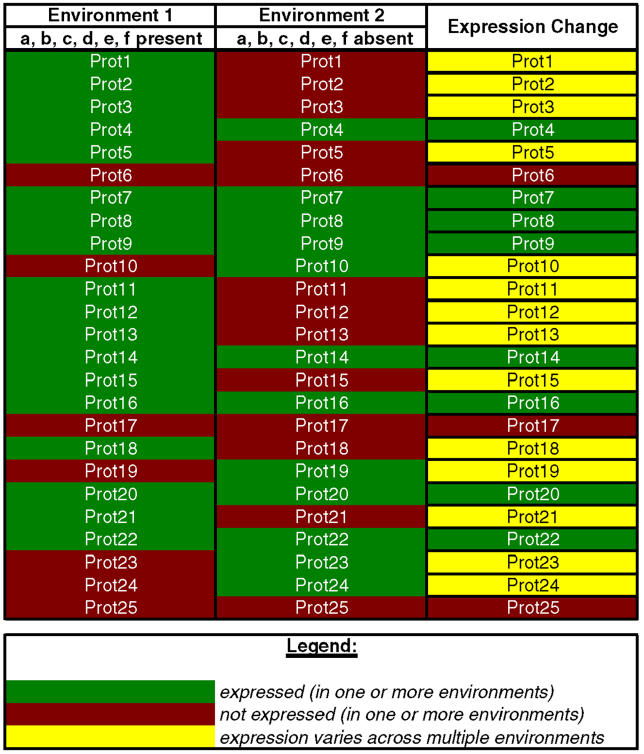
In Silico Expression Array for the Prototypic TRS The expression of genes is evaluated across two different environments using extreme pathway analysis. The first column depicts the expression state for an environment in which all six metabolites are present. The second column depicts the expression state for an environment in which all six metabolites are absent. For both columns, the proteins shaded in green are transcribed, whereas those in red are not transcribed. The third column illustrates an in silico expression analysis: the expression states of the two environments are compared, and changes in expression are illustrated in yellow, as described in the legend box on the figure.

Furthermore, the basis vectors that result from extreme pathway analysis effectively produce an in silico expression array. Figure 11 illustrates how two possible environments may be compared. For example, Protein 1 is not expressed when the six extracellular metabolites are absent, yet it is expressed when they are present. By contrast, Protein 4 is expressed in both of these environments, i.e., when the six extracellular metabolites are all absent as well as when they are all present. These in silico expression analyses are faster and cheaper than experimental arrays, and they can provide novel hypotheses and consequently serve as starting points for further experimental work.

#### The left null space of R*.

The basis vectors for the left null space of the matrix of the prototypic system identified intra-network pools in the TRS. For the case of the prototypic system, these were expected groupings of the genes and gene products. For example, one such intra-network pool consisted of Protein 14*,* Protein 20*,* and Protein 22. The expression of Protein 20 requires the presence of Protein 14. Similarly, the expression of Protein 22 requires the presence of Protein 14. Consequently, these proteins comprise a single pool within the TRS that is coordinately regulated as a single regulated unit. For TRSs of a larger scale as is seen in actual biological systems on the order of the genome-scale reconstruction of *E. coli,* more complex groupings may emerge.

#### The row and column spaces of R*.

The row and column spaces were calculated for the prototypic system (unpublished data). The data indicated patterns as described in the Materials and Methods section above. Further investigation into the row and column spaces of genome-scale TRSs may generate predictions regarding optimal experimental programs for characterizing regulatory programs as described above. Such interrogations may also reveal how a TRS moves from one expression state to another given environmental perturbations and genetic modifications.

## Discussion

This study describes a matrix formalism for studying TRSs that connects environmental cues to transcriptional responses. The TRS of the *lac* operon in E. coli was described. Furthermore, the TRS for a prototypic system that mimics features of the E. coli TRS was characterized and the fundamental subspaces of the corresponding R* matrix were described. Key results of this study are: 1) a systems definition and approach to TRNs, distinguishing between a TRS and a TRN; 2) the matrix formalization of a TRS as an alternative to a Boolean formalism; 3) the characterization of the null and left null spaces of R*; and 4) the exhaustive enumeration of the effect of all possible environments and consequent systemic interpretations.

The formalism presented herein is a conceptual shift in the representation of a TRN (Figure 1). While a network map illustrates relationships between components of a network, it does not allow for the computation of functional states. However, TRNs can be represented mathematically with the quasi-stoichiometric formalism presented herein. With careful delineation of inputs and outputs, a TRS is defined from which functional states can be computed.

There is a growing set of computational tools and approaches for the analysis of biological systems. For example, stoichiometric matrices are analyzed with flux-balance analysis, extreme pathway analysis [25], Monte Carlo sampling [16], and energy balance analysis [36], among many other such computational tools [16]. These analysis methods generate unbiased descriptions of the functional states of biochemical networks. As TRSs are reconstructed with a matrix formalism as presented herein, these analysis tools can be used to characterize fundamental features of such systems.

Key properties of the TRS are found in the two null spaces of R*. As stated in the description of the quasi-stoichiometric formalism above, the null space of R* represents the set of causality pathways that connect given environments to the expression state of the TRS, accounting for the primary, secondary, and tertiary regulatory relationships. This null space can be described by a unique set of link-neutral pathways in which all regulatory rules or links are balanced. This balanced set of pathways can be calculated with extreme pathway analysis which characterizes the extreme pathways of the given TRS from which all expression (i.e., functional) states can be described. The left null space of R* contains the pools of invariant quantities of the TRS. These invariant pools represent transcriptional units in the given system that may generate hypotheses regarding correlated regulatory programs. Extreme pathway analysis can also be used to calculate these invariant pools by defining the node-neutral set of extreme states.

All possible expression states of the prototypic TRS are readily evaluated. This data allows for the classification of the percentage of environments in which a given gene is expressed as well as the average number of genes expressed in a set of environments. This analysis clearly delineates the genes that are generally active or generally inactive across a variety of environments and perhaps more critical or less critical for network function. For larger TRSs, sampling algorithms can be implemented to perform similar analyses [21]. Characterizing the space of possible expression states also leads to the identification of correlated gene sets. These correlations identify how “related” the expression profiles are for the associated pairs, and can generate hypotheses regarding operon or regulon structure. These correlations also identify genes that are anticorrelated or that behave independently. By analyzing all possible expression states of a TRS, such systems-level properties are readily described.

The regulatory network matrices presented herein are in quasi-stoichiometric formalism. As more data emerges regarding specific chemical transformations and interactions that define TRSs, increasingly detailed matrices to include the precise underlying reaction stoichiometry may be generated. The analysis of the *lac* operon network presented herein led to results representative of what may be seen in genome-scale analyses. The analysis of the stoichiometric matrix for the set of regulatory mechanism reactions (e.g., the binding of a transcription factor and associated regulatory proteins to a specific region of DNA) will reveal much greater quantitative detail regarding TRSs [17]. However, the necessary data is only now becoming available. The matrix formalism presented herein is a structured method for organizing the set of hypotheses regarding TRS function. For example, additional novel regulatory rules for the expression of genes can be added to the matrix as they are characterized and existing rules can be refined as the specific reaction stoichiometries are more clearly defined. Any change in network properties resulting from the inclusion of new rules can be verified to support or refute the given hypothesis. Furthermore, as greater stoichiometric detail becomes available, this framework readily incorporates the associated data. If one regulatory reaction is better characterized, the associated quasi-stoichiometric reaction is replaced with the stoichiometric detail (the corresponding column of **R** is replaced).

Previous work characterized essential nodes for the processing of signaling inputs to TRNs [37]. These subnetworks, called “origons,” are believed to represent specific topological units of TRNs that detect the decomposed elementary components of complex environmental signals and subsequently develop a reassembled, large-scale transcriptional response. Furthermore, “network motifs,” or patterns of interconnections that recur in many different parts of a network at frequencies higher than those found in randomized networks, have been evaluated, and may define basic building blocks of TRNs [38]. The study described herein differs from these previous works because it focuses on the structure of an entire TRS that is reconstructed by delineating functional relationships between genes and inputs. As such, the characterizations described herein are more directly connected to network function as opposed to network structure.

With this formalism in hand, the challenge now becomes to scale it up and construct genome-scale TRS matrices for model organisms, such as E. coli and Bacillus subtilis. With these matrices in hand, experimental programs can be systematized and guided with predictions regarding which signaling inputs may provide the greatest characterization of network function. Furthermore, these analyses will generate predictions regarding the coordinated regulatory programs that drive cellular phenotypes.

## Supporting Information

Protocol S1 Stoichiometric Network Reconstruction and Associated Analyses Techniques Found at DOI: 10.1371/journal.pcbi.0020101.sd001 (58 KB DOC)

Protocol S2 Characteristics of the Prototypic TRS Found at DOI: 10.1371/journal.pcbi.0020101.sd002 (106 KB PDF)

## Abbreviations

cAMP: cyclic AMP
TRN: transcriptional regulatory network
TRS: transcriptional regulatory system
