# Supplementary material for: Matrix Formalism to Describe Functional States of Transcriptional Regulatory Systems
Source: PLoS Comput Biol. 2006 Aug 11;2(8):e101. doi: 10.1371/journal.pcbi.0020101 (PMC1534074; doi:10.1371/journal.pcbi.0020101)
Supplement: Supplementary file 1 [file pcbi.0020101.sd002.pdf]

PROTOCOL 2

In an effort to investigate the conceptual framework and associated results, a prototypic transcriptional regulatory network (TRN) was assembled and evaluated. The prototypic TRN is illustrated in Figure S1(A). The Boolean expressions that describe the regulatory rules for gene transcription appear in Figure S1(B). The prototypic TRN used in this study was composed of regulatory rules associated with the expression of 25 genes. These rules were typical of Boolean rules in the *E. coli* reconstruction published previously [1]. The given regulatory rule must be satisfied in its entirety for the corresponding gene to be expressed; otherwise, the gene is not expressed. The complete transcriptional regulatory system (TRS) accounted for the presence or absence of six compounds (*a* through *f*) and five protein products that acted as "transcription factors" for other genes (*Prot 1, Prot 6, Prot 8, Prot 11*, and *Prot 14*). Here we describe basic properties of the prototypic TRN and the corresponding TRS that accounts for inputs and outputs of the TRN (Figure 1(C)), as well as the expression (i.e., functional) states computed from the matrix formalization of the prototypic TRS.

Characteristics of the prototypic TRN

Since the prototypic TRN was constructed on the basis of regulatory rules observed in the integrated *E. coli* regulatory and metabolic network [1], it was comprised of both primary and secondary regulatory relationships. For example, the expression of *Gene 1* is an example of a primary regulatory relationship, since it requires the presence of *Metabolite a* and *Metabolite b*. A secondary regulatory relationship, meanwhile, is seen in the rule for *Gene 12*; its expression requires the presence of the protein product of *Gene 1*. Ultimately, the prototypic TRN consisted of three levels of transcription, as observed in the integrated *E. coli* regulatory and metabolic network. Such a three-tiered cascade of transcriptional events is exemplified by the set of regulatory rules for *Gene 1*, *Gene 14*, and *Gene 20* in the prototypic system. Certain extra-cellular cues affect the expression of *Gene 1*; *Protein 1*, the product of *Gene 1*, is a downstream transcription factor for *Gene*

14; and *Protein 14* affects the expression of *Gene 20*. The matrix formalism described in the manuscript provides a natural format for delineating these types of relationships.

The prototypic TRN also included genes whose regulatory rules imply operon and regulon structures [2, 3]. As an example of operon representation, *Gene 1*, *Gene 2*, and *Gene 3* are modeled to be physically located adjacent to one another (Figure S1(A)), and are co-expressed since they all require the presence of metabolites *a* and *b* in order to be transcribed. As a representative regulon, *Gene 20* and *Gene 22* are also co-expressed, but these genes are represented at different locations within the genome (Figure S1(B)).

Furthermore, we maintained the number of environmental cues per target gene at about 2. In general, the number of inputs affecting a target gene should average on the order of 2 in order to ensure network stability while allowing for evolutionary improvements [4]. Furthermore, the number of total inputs to the system versus the number of total genes regulated within the system was set at about 0.4, as is the case for *E. coli*. Likewise, the ratio of the number of extracellular metabolites to the number of transcription factors was set at about 1.

A regulatory network matrix **R** was constructed for the prototypic TRS and captured the regulatory rules for the 25 genes of the TRN in the quasi-stoichiometric formalism described in the manuscript. The size of this matrix was 62 rows [= (2 x 6 metabolites) + (2 x 25 genes)] by 101 columns [= (39 activation reactions) + (37 inhibition reactions) + (25 protein exchange reactions)]. Furthermore, this matrix was combined with each of the 64 possible environment matrices (**E**) [= 2^6, where there are six inputs], each of size 62 rows [= (2 x availability of 6 metabolites) + (presence of 25 genes) + (absence of 25 genes)] by 31 columns [= (availability of 6 metabolites) + (availability of 25 protein products)], and the fundamental subspaces of the resultant adjacency matrices **R*** were analyzed to assess network characteristics.

Connectivity properties of the prototypic TRS calculated using ***R***

Two features of **R** provide insight into the degree of connectivity in the regulatory network. First, the number of inputs, including extra-cellular metabolites and internally-produced transcription factors, that are evaluated for a given gene indicates the degree of complexity in the associated regulatory rule. This feature was evaluated for the prototypic network (Figure S2(A)). The results were divided into two groups: (1) the number of inputs whose *presence* is critical for the expression of the associated gene (shown as stars), and (2) the number of inputs whose *absence* is critical for the expression of the associated gene (shown as open circles). For example, the regulatory rule associated with expression of *Gene 7* involves the evaluation of whether two different compounds are present and a third is absent (indicated by the arrow). The scope of the division between present and absent input signals in the regulatory network matrix may characterize the relative importance of transcriptional activators versus inhibitors in actual TRSs.

Second, the number of genes in which a given input is evaluated indicates the relative pervasiveness of a given signaling input in affecting the TRN. (For example, the presence of oxygen participates in the regulatory rules associated with a large number of genes in energy metabolism.) This feature was evaluated for the prototypic TRS, both for the extracellular metabolites and the internally-produced transcription factors (Figures S2(B) and S2(C)). The presence of *Metabolite b* is important for the expression of four genes, whereas the absence of this metabolite is important for the expression of one gene (indicated by the arrow in Figure S2(B)). The presence of *Protein 8*, the protein product of *Gene 8* within the TRS, is important for the expression of four genes, whereas the absence of this protein is important for the expression of two genes (indicated by the arrow in Figure S2(C)). These statistics clearly indicate compounds and regulators that generate the most widespread effect on gene expression for TRSs and therefore may suggest good targets for future experimental designs.

*Functional states calculated using **R****

The evaluation of all possible environments (all possible combinations of inputs) facilitated the identification and analysis of the properties of the prototypic TRS shown in Figure S1. As described in the manuscript, extreme pathways are convex basis vectors of the null space of a matrix that satisfy constraints which ensure that the associated pathways are biologically relevant. These extreme pathways correspond to basis vectors that represent the extreme states of the TRS; any possible expression state of a TRS (i.e., corresponding to a given environment) is a non-negative combination of these basis vectors.

There were 133 unique extreme pathways for the prototypic TRS. These extreme pathways are shown in Figure S3. These pathways were obtained by evaluating the null space of each of the 64 possible adjacency matrices (**R***), one for each of the 64 possible environments (**E**). Thus the resultant extreme pathways can be grouped together to form all possible expression states of the prototypic TRS.

FIGURE CAPTIONS

Fig. S1. A prototypic TRS. In panel (A), a prototypic TRS consisting of 25 genes, six extracellular metabolites, and five transcription factors, is shown. This prototype was constructed on the basis of the general characteristics of the *E. coli* TRS. In panel (B), the Boolean regulatory rules that correspond to the transcription of the 25 genes within the prototypic TRN are listed. Relevant characteristics of the prototypic TRS, including comparisons to the regulatory network of *E. coli*, are presented in panel (C). This prototypic TRS gives rise to 64 [= 2^6, where there are six inputs] possible environments, and these may be evaluated using the 64 different adjacency matrices that are generated by combining the TRS **R** matrix with each of the 64 different environment matrices (**E**).

Fig. S2. The pervasiveness of signal inputs in the prototypic TRS. In panel (A), the number of inputs (*y*-axis) that affect the expression of the given gene (*x*-axis) is indicated. For example, the rule associated with the expression of *Gene 7* evaluates the presence of two inputs (asterisk) and the absence (open circle) of one input (as indicated by the

arrow). Panel (B) depicts the number of genes (*y*-axis) whose expression is affected by the given input. Similarly, in panel (C), the number of genes (*y*-axis) whose expression is affected by the given protein is indicated. For example, the presence of compound *b* (asterisk) affects the expression of four genes, whereas the absence of compound *b* (open circle) affects the expression of one gene (as indicated by the arrow in panel (B)); and the presence of *Protein 14* (asterisk) affects the expression of four genes, whereas the absence of *Protein 14* (open circle) affects the expression of two genes (as indicated by the arrow in panel (C)). Note that these graphs are rank-ordered by presence.

Fig. S3. The extreme pathways for the prototypic TRS. Extreme pathway analysis of **R*** yields the expression (i.e., functional) states. The 133 extreme pathways for **R*** of the prototypic TRS are presented. Rows shaded in light green denote routes of activation of gene expression, whereas rows shaded in light red denote routes of inactivation of gene expression. These extreme pathways collectively represent all possible expression (i.e., functional) states across all possible environments, i.e., all possible combinations of the presence and absence of inputs to the system. In other words, a combination of the extreme pathways (a subset of the 133 extreme pathways for the prototypic TRS) corresponds to the expression state for a given environment, which is defined by the activity (i.e., the presence or absence) of the extracellular cues to the TRN. Consequently, for any gene, there exist multiple pathways leading to activation or inactivation of gene expression.

REFERENCES

1. Covert, M.W., *et al.* (2004) Integrating high-throughput and computational data elucidates bacterial networks. *Nature* 429, 92-96
2. Alberts, B. (2002) *Molecular biology of the cell*. Garland Science
3. Shen-Orr, S.S., *et al.* (2002) Network motifs in the transcriptional regulation network of Escherichia coli. *Nat Genet* 31, 64-68
4. Kauffman, S.A. (1993) *The origins of order: self-organization and selection in evolution*. Oxford University Press

Figure S1

A

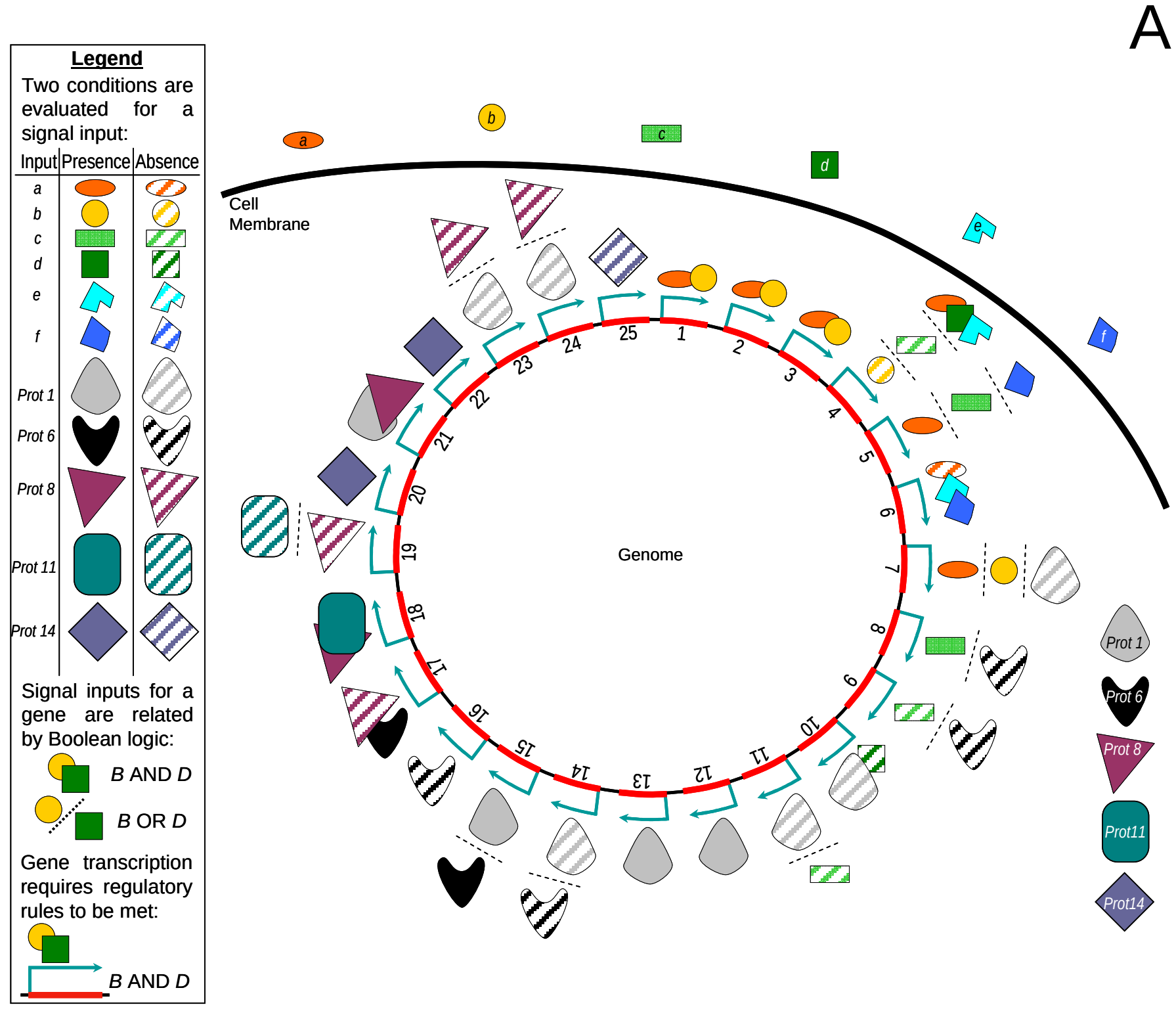

B

Genes	Rules
Gene 1	a AND b
Gene 2	a AND b
Gene 3	a AND b
Gene 4	NOT(b) OR NOT(c) OR (a AND d AND e)
Gene 5	a OR c OR f
Gene 6	NOT(a) AND e AND f
Gene 7	(a OR b) OR NOT(Prot 1)
Gene 8	c OR NOT(Prot 6)
Gene 9	NOT(c AND Prot 6)
Gene 10	NOT(d OR Prot 1)
Gene 11	Prot 1 OR c
Gene 12	Prot 1
Gene 13	Prot 1
Gene 14	NOT(Prot 1 AND Prot 6)
Gene 15	Prot 1 OR Prot 6
Gene 16	NOT(Prot 6)
Gene 17	Prot 6 AND NOT(Prot 8)
Gene 18	Prot 8 AND Prot 11
Gene 19	NOT(Prot 8 AND Prot 11)
Gene 20	Prot 14
Gene 21	Prot 1 AND Prot 8
Gene 22	Prot 14
Gene 23	NOT(Prot 1 AND Prot 8)
Gene 24	NOT(Prot 1 AND Prot 8)
Gene 25	NOT(Prot 14)

C

Network Characteristics	
Number of genes	25
Metabolites	6
Transcription factors	5
Levels of transcription	3
Possible environments	64 [$=2^6$]

Comparison to *E. coli*	Prototype	*E. coli*
Input to gene ratio	0.44	0.36
Metabolites to transcription factor	1.20	1.00
Inputs per gene (k_{in})	2.00	2.00

Figure S2

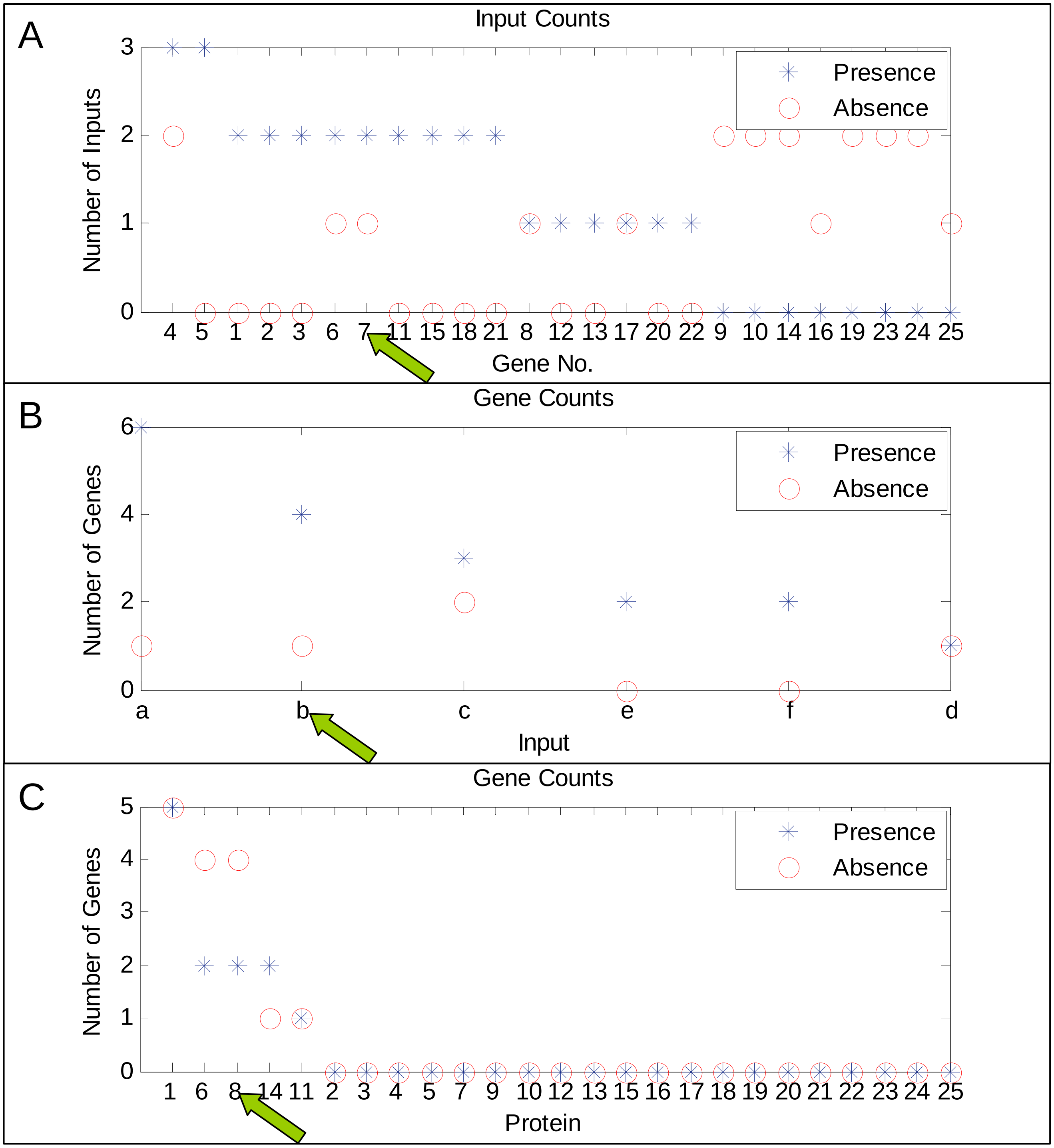
A
Input Counts
Number of Inputs
0
1
2
3
4 5 1 2 3 6 7 11 15 18 21 8 12 13 17 20 22 9 10 14 16 19 23 24 25
Gene No.
Presence
Absence
B
Gene Counts
Number of Genes
0
2
4
6
a b c e f d
Input
Presence
Absence
C
Gene Counts
Number of Genes
0
1
2
3
4
5
1 6 8 14 11 2 3 4 5 7 9 10 12 13 15 16 17 18 19 20 21 22 23 24 25
Protein
Presence
Absence

Figure S3

Pathway leading to activation or inactivation of gene	Gene no.
Gene1, a, b, Prot1	1
not_Gene1a, not_a, not_Prot1	1
not_Gene1b, not_b, not_Prot1	1
Gene2, a, b, Prot2	2
not_Gene2b, not_b, not_Prot2	2
not_Gene2a, not_a, not_Prot2	2
Gene3, a, b, Prot3	3
not_Gene3b, not_b, not_Prot3	3
not_Gene3a, not_a, not_Prot3	3
Gene4b, not_c, Prot4	4
Gene4a, not_b, Prot4	4
Gene4c, a, d, e, Prot4	4
not_Gene4b, b, c, not_d, not_Prot4	4
not_Gene4c, b, c, not_e, not_Prot4	4
not_Gene4a, b, c, not_a, not_Prot4	4
Gene5a, a, Prot5	5
Gene5c, f, Prot5	5
Gene5b, c, Prot5	5
not_Gene5, not_a, not_c, not_f, not_Prot5	5
Gene6, e, f, not_a, Prot6	6
not_Gene6b, not_e, not_Prot6	6
not_Gene6c, not_f, not_Prot6	6
not_Gene6a, a, not_Prot6	6
not_Gene1a, Gene7c, not_a, Prot7	7
not_Gene1b, Gene7c, not_b, Prot7	7
Gene7b, b, Prot7	7
Gene7a, a, Prot7	7
Gene8a, c, Prot8	8
not_Gene6b, Gene8b, not_e, Prot8	8
not_Gene6c, Gene8b, not_f, Prot8	8
not_Gene6a, Gene8b, a, Prot8	8
Gene6, not_Gene8, e, f, not_a, not_c, not_Prot8	8
Gene9a, not_c, Prot9	9
not_Gene6b, Gene9b, not_e, Prot9	9
not_Gene6c, Gene9b, not_f, Prot9	9
not_Gene6a, Gene9b, a, Prot9	9
Gene6, not_Gene9, c, e, f, not_a, not_Prot9	9
not_Gene1a, Gene10, not_a, not_d, Prot10	10
not_Gene1b, Gene10, not_b, not_d, Prot10	10
Gene1, not_Gene10b, a, b, not_Prot10	10
not_Gene10a, d, not_Prot10	10
Gene1, Gene11a, a, b, Prot11	11
Gene11b, c, Prot11	11
not_Gene1a, not_Gene11, not_a, not_c, not_Gene11	11
not_Gene1b, not_Gene11, not_b, not_c, not_Gene11	11
Gene1, Gene12, a, b, Prot12	12
not_Gene1a, not_Gene12, not_a, not_Prot12	12
not_Gene1b, not_Gene12, not_b, not_Prot12	12
Gene1, Gene13, a, b, Prot13	13
not_Gene1a, not_Gene13, not_a, not_Prot13	13
not_Gene1b, not_Gene13, not_b, not_Prot13	13
not_Gene1a, Gene14a, not_a, Prot14	14
not_Gene1b, Gene14a, not_b, Prot14	14
not_Gene6b, Gene14b, not_e, Prot14	14
not_Gene6c, Gene14b, not_f, Prot14	14
not_Gene6a, Gene14b, a, Prot14	14
Gene6, Gene15b, e, f, not_a, Prot15	15
Gene1, Gene15a, a, b, Prot15	15
not_Gene1b, not_Gene6b, not_Gene15, not_b, not_e, not_Prot15	15
not_Gene1a, not_Gene6b, not_Gene15, not_a, not_e, not_Prot15	15
not_Gene1b, not_Gene6c, not_Gene15, not_b, not_f, not_Prot15	15
not_Gene1a, not_Gene6c, not_Gene15, not_a, not_f, not_Prot15	15
not_Gene1b, not_Gene6a, not_Gene15, a, not_b, not_Prot15	15
not_Gene6b, Gene16, not_e, Prot16	16
not_Gene6c, Gene16, not_f, Prot16	16
not_Gene6a, Gene16, a, Prot16	16
Gene6, not_Gene16, e, f, not_a, not_Prot16	16

Pathway leading to activation or inactivation of gene	Gene No.
not_Gene8, Gene17, (2)e, (2)f, (2) not_a, not_c, Prot17	17
Gene8a, not_Gene17b, c, not_Prot17	17
not_Gene6b, Gene8b, not_Gene17b, not_e, not_Prot17	17
not_Gene6b, not_Gene17a, not_e, not_Prot17	17
not_Gene6c, Gene8b, not_Gene17b, not_f, not_Prot17	17
not_Gene6c, not_Gene17a, not_f, not_Prot17	17
not_Gene6a, Gene8b, not_Gene17b, a, not_Prot17	17
not_Gene6a, not_Gene17a, a, not_Prot17	17
Gene1, Gene8a, Gene11a, Gene18, a, b, c, Prot18	18
Gene8a, Gene11b, Gene18, 2(c), Prot18	18
Gene1, not_Gene6b, Gene8b, Gene11a, Gene18, a, b, not_e, Prot18	18
not_Gene6b, Gene8b, Gene11b, Gene18, c, not_e, Prot18	18
Gene1, not_Gene6c, Gene8b, Gene11a, Gene18, a, b, not_f, Prot18	18
not_Gene6c, Gene8b, Gene11b, Gene18, c, not_f, Prot18	18
Gene1, not_Gene6a, Gene8b, Gene11a, Gene18, (2)a, b, Prot18	18
not_Gene6a, Gene8b, Gene11b, Gene18, a, c, Prot18	18
Gene6, not_Gene8, not_Gene18a, e, f, not_a, not_c, not_Prot18	18
not_Gene1a, not_Gene11, not_Gene18b, not_a, not_c, not_Prot18	18
not_Gene1b, not_Gene11, not_Gene18b, not_b, not_c, not_Prot18	18
not_Gene1a, not_Gene11, Gene19b, not_a, not_c, Prot19	19
Gene6, not_Gene8, Gene19a, e, f, not_a, not_c, Prot19	19
not_Gene1b, not_Gene11, Gene19b, not_b, not_c, Prot19	19
Gene1, Gene8a, Gene11a, not_Gene19, a, b, c, not_Prot19	19
Gene8a, Gene11b, not_Gene19, (2)c, not_Prot19	19
Gene1, not_Gene6b, Gene8b, Gene11a, not_Gene19, a, b, not_e, not_Prot19	19
not_Gene6b, Gene8b, Gene11b, not_Gene19, c, not_e, not_Prot19	19
Gene1, not_Gene6c, Gene8b, Gene11a, not_Gene19, a, b, not_f, not_Prot19	19
not_Gene6c, Gene8b, Gene11b, not_Gene19, c, not_f, not_Prot19	19
Gene1, not_Gene6a, Gene8b, Gene11a, not_Gene19, (2)a, b, not_Prot19	19
not_Gene6a, Gene8b, Gene11b, not_Gene19, a, c, not_Prot19	19
not_Gene1a, Gene14a, Gene20, not_a, Prot20	20
not_Gene1b, Gene14a, Gene20, not_b, Prot20	20
not_Gene6b, Gene14b, Gene20, not_e, Prot20	20
not_Gene6c, Gene14b, Gene20, not_f, Prot20	20
not_Gene6a, Gene14b, Gene20, a, Prot20	20
Gene1, Gene8a, Gene21, a, b, c, Prot21	21
Gene1, not_Gene6b, Gene8b, Gene21, a, b, not_e, Prot21	21
Gene1, not_Gene6c, Gene8b, Gene21, a, b, not_f, Prot21	21
Gene1, not_Gene6a, Gene8b, Gene21, (2)a, b, Prot21	21
Gene6, not_Gene8, not_Gene21b, e, f, not_a, not_c, not_Prot21	21
not_Gene1a, not_Gene21a, not_a, not_Prot21	21
not_Gene1b, not_Gene21a, not_b, not_Prot21	21
not_Gene1a, Gene14a, Gene22, not_a, Prot22	22
not_Gene1b, Gene14a, Gene22, not_b, Prot22	22
not_Gene6b, Gene14b, Gene22, not_e, Prot22	22
not_Gene6c, Gene14b, Gene22, not_f, Prot22	22
not_Gene6a, Gene14b, Gene22, a, Prot22	22
not_Gene1a, Gene23a, not_a, Prot23	23
not_Gene1b, Gene23a, not_b, Prot23	23
Gene6, not_Gene8, Gene23b, e, f, not_a, not_c, Prot23	23
Gene1, Gene8a, not_Gene23, a, b, c, not_Prot23	23
Gene1, not_Gene6b, Gene8b, not_Gene23, a, b, not_e, not_Prot23	23
Gene1, not_Gene6c, Gene8b, not_Gene23, a, b, not_f, not_Prot23	23
Gene1, not_Gene6a, Gene8b, not_Gene23, 2(a), b, not_Prot23	23
not_Gene1a, Gene24a, not_a, Prot24	24
not_Gene1b, Gene24a, not_b, Prot24	24
Gene6, not_Gene8, Gene24b, e, f, not_a, not_c, Prot24	24
Gene1, Gene8a, not_Gene24, a, b, c, not_Prot24	24
Gene1, not_Gene6b, Gene8b, not_Gene24, a, b, not_e, not_Prot24	24
Gene1, not_Gene6c, Gene8b, not_Gene24, a, b, not_f, not_Prot24	24
Gene1, not_Gene6a, Gene8b, not_Gene24, 2(a), b, not_Prot24	24
not_Gene1a, Gene14a, not_Gene25, not_a, not_Prot25	25
not_Gene1b, Gene14a, not_Gene25, not_b, not_Prot25	25
not_Gene6b, Gene14b, not_Gene25, not_e, not_Prot25	25
not_Gene6c, Gene14b, not_Gene25, not_f, not_Prot25	25
not_Gene6a, Gene14b, not_Gene25, a, Prot25	25
