# Supplementary material for: Matrix Formalism to Describe Functional States of Transcriptional Regulatory Systems
Source: PLoS Comput Biol. 2006 Aug 11;2(8):e101. doi: 10.1371/journal.pcbi.0020101 (PMC1534074; doi:10.1371/journal.pcbi.0020101)
Supplement: Supplementary file 2 [file pcbi.0020101.sd001.doc]

**PROTOCOL 1**

High-throughput experimental data coupled with mathematical modeling enables the identification of novel hypotheses and emergent properties of biological systems. A stoichiometric matrix captures a detailed description of a biochemical network and forms the basis for subsequent analyses of fundamental network characteristics such as crosstalk, redundancy, and modularity. Indeed, the stoichiometric reconstruction and associated analyses have been applied with success to metabolic and signaling networks [1]. In this work, we attempt to expand upon this formalism by applying to transcriptional regulatory systems (TRSs) a modified, quasi-stoichiometric formalism that takes into account all underlying component relationships but is not necessarily mass-balanced. Importantly, however, this formalism can account for mass-balanced relationships as they become delineated for real TRSs. Here we provide background regarding the stoichiometric network reconstruction and associated analyses, as previously applied to metabolic and signaling networks.

The stoichiometric reconstruction is effectively a catalog of all the chemical transformations that take place within a system of defined boundaries, as assimilated through the careful integration of experimental data [2]. Specifically, experimental data on the components of a network and the associated interactions between these components is synthesized into a matrix form, often called the stoichiometric matrix or **S**. The rows of **S** correspond to the components of the network (i.e., individual chemical species such as proteins and metabolites), and the columns correspond to the chemical transformations or interactions among the components (i.e., individual reactions). The elements of **S** correspond to the stoichiometric coefficients of the associated chemical transformations. By definition, these coefficients are biologically universal, i.e., they are not variable as functions of temperature, pH, or other environmental conditions. In a given reaction in **S**, if a particular component is formed by the reaction, the corresponding stoichiometric coefficient has a positive sign, and if it is consumed by the reaction, the corresponding stoichiometric coefficient has a negative sign. All other rows, i.e., those corresponding to components that do not participate in the reaction, are zero. Consequently, the stoichiometric matrix provides a mathematical formalism to account for interactions of components throughout a biochemical network.

Several mathematical approaches derived from matrix algebra can be utilized to analyze properties of the reconstruction. “Constraint-based” approaches involve the mathematical description of physico-chemical constraints to which a cellular network is subject [3]. One such approach used in this manuscript is referred to as network-based pathway analysis [4-6]. This technique effectively involves the calculation of basis vectors of the stoichiometric matrix that have biochemical meaning. This topic has been extensively reviewed [4-6].

**REFERENCES**

1.	Papin, J.A., and Palsson, B.O. (2004) The JAK-STAT signaling network in the human B-cell: an extreme signaling pathway analysis. *Biophys J* 87, 37-46

2.	Gianchandani, E.P.*, et al.* (2006) Systems analyses characterize integrated functions of biochemical networks. *Trends Biochem Sci* 31, 284-291

3.	Price, N.D.*, et al.* (2004) Genome-scale models of microbial cells: evaluating the consequences of constraints. *Nat Rev Microbiol* 2, 886-897

4.	Papin, J.A.*, et al.* (2004) Comparison of network-based pathway analysis methods. *Trends Biotechnol* 22, 400-405

5.	Papin, J.A.*, et al.* (2003) Metabolic pathways in the post-genome era. *Trends Biochem Sci* 28, 250-258

6.	Schilling, C.H.*, et al.* (2000) Theory for the systemic definition of metabolic pathways and their use in interpreting metabolic function from a pathway-oriented perspective. *J. Theor. Biol.* 203, 229-248.
